# Empowering standardization of cancer vaccines through ontology: enhanced modeling and data analysis

**DOI:** 10.1186/s13326-024-00312-3

**Published:** 2024-06-19

**Authors:** Jie Zheng, Xingxian Li, Anna Maria Masci, Hayleigh Kahn, Anthony Huffman, Eliyas Asfaw, Yuanyi Pan, Jinjing Guo, Virginia He, Justin Song, Andrey I. Seleznev, Asiyah Yu Lin, Yongqun He

**Affiliations:** 1 Unit for Laboratory Animal Medicine, University of Michigan, Ann Arbor, MI 48109 USA; 2 College of Literature, Science, and the Arts, University of Michigan, Ann Arbor, MI 48109 USA; 3 Data Impact and Governance, Technology Data and Innovation, University of Texas MD Anderson Cancer Center, Houston, TX 77030 USA; 4 Department of Computational Medicine and Bioinformatics, University of Michigan Medical School, Ann Arbor, MI 48109 USA; 5 University of Michigan Medical School, Ann Arbor, MI 48109 USA; 6 The College of Brown University, Brown University, Providence, RI 02912 USA; 7 College of Electrical Engineering and Computer Science, University of Michigan, Ann Arbor, MI 48109 USA; 8 Dietrich School of Arts and Sciences, University of Pittsburgh, Pittsburgh, PA 15260 USA; 9 Axle Research and Technology, Rockville, MD 20852 USA; 10 Rogel Cancer Center, University of Michigan Medical School, Ann Arbor, MI 48109 USA

**Keywords:** Cancer vaccine, Vaccine ontology, Ontology design pattern, Ontology modeling, CanVaxKB

## Abstract

**Background:**

The exploration of cancer vaccines has yielded a multitude of studies, resulting in a diverse collection of information. The heterogeneity of cancer vaccine data significantly impedes effective integration and analysis. While CanVaxKB serves as a pioneering database for over 670 manually annotated cancer vaccines, it is important to distinguish that a database, on its own, does not offer the structured relationships and standardized definitions found in an ontology. Recognizing this, we expanded the Vaccine Ontology (VO) to include those cancer vaccines present in CanVaxKB that were not initially covered, enhancing VO’s capacity to systematically define and interrelate cancer vaccines.

**Results:**

An ontology design pattern (ODP) was first developed and applied to semantically represent various cancer vaccines, capturing their associated entities and relations. By applying the ODP, we generated a cancer vaccine template in a tabular format and converted it into the RDF/OWL format for generation of cancer vaccine terms in the VO. ‘12MP vaccine’ was used as an example of cancer vaccines to demonstrate the application of the ODP. VO also reuses reference ontology terms to represent entities such as cancer diseases and vaccine hosts. Description Logic (DL) and SPARQL query scripts were developed and used to query for cancer vaccines based on different vaccine’s features and to demonstrate the versatility of the VO representation. Additionally, ontological modeling was applied to illustrate cancer vaccine related concepts and studies for in-depth cancer vaccine analysis. A cancer vaccine-specific VO view, referred to as “CVO,” was generated, and it contains 928 classes including 704 cancer vaccines. The CVO OWL file is publicly available on: http://purl.obolibrary.org/obo/vo/cvo.owl, for sharing and applications.

**Conclusion:**

To facilitate the standardization, integration, and analysis of cancer vaccine data, we expanded the Vaccine Ontology (VO) to systematically model and represent cancer vaccines. We also developed a pipeline to automate the inclusion of cancer vaccines and associated terms in the VO. This not only enriches the data’s standardization and integration, but also leverages ontological modeling to deepen the analysis of cancer vaccine information, maximizing benefits for researchers and clinicians.

**Availability:**

The VO-cancer GitHub website is: https://github.com/vaccineontology/VO/tree/master/CVO.

## Background

Cancer, with its myriad forms and insidious nature, has long been one of the most formidable adversaries in the realm of medicine. Traditional treatments such as chemotherapy, surgery, and radiation therapy have been the cornerstone of cancer care, offering varying degrees of success in combating the disease [1–3]. However, the limitations and side effects associated with these treatments have spurred a quest for more targeted and effective therapeutic approaches [4, 5]. The first immunotherapy for cancer is “Coley’s toxin” developed by William Coley, which stimulates the antitumor immune system response [6]. Since then, cancer vaccines, as a form of immunotherapy, have been increasingly studied to treat various cancers. Some cancer vaccines, such as Sipuleucel-T (Provenge) [7, 8], human papillomavirus (HPV) vaccine [9–11], have been approved by the U.S. Food and Drug Administration (FDA) and demonstrated notable success. However, there are a very large number of cancer vaccines in the development stage, most of which are in the pre-clinical trial and clinical trial stage [12–14]. Compared to traditional treatments, cancer vaccines operate by harnessing the body’s immune system to recognize and destroy cancer cells [15]. Thus, cancer vaccines have the advantage of offering a more targeted and systemic approach, sparing healthy tissues while selectively targeting cancer cells. This precision not only minimizes the risk of adverse effects but also opens up new possibilities for combination therapies that synergistically enhance treatment efficacy [5, 16].

Cancer vaccines are fundamentally divided into two principal categories: preventive and therapeutic. Preventive vaccines, exemplified by those targeting HPV [17, 18] and Hepatitis B [19], are designed to prevent cancer development associated with viral infections by stimulating an immune response against the virus itself. By targeting specific cancer antigens or mutations, vaccines can train the immune system to recognize and eliminate precancerous cells before they develop into full-blown tumors. In contrast, therapeutic cancer vaccines aim to treat existing cancers by boosting the immune system’s capacity to identify and destroy cancer cells using tumor antigens [12, 14]. However, the intricate mechanisms underlying cancer vaccines are not fully understood, necessitating a comprehensive representation of their complexities and associated information. With the increasing amount of data existing, it is critical to represent complex cancer vaccines and their associated information.

CanVaxKB (https://violinet.org/canvaxkb) is the first web-based cancer vaccine knowledgebase that has compiled > 670 therapeutic or preventive cancer vaccines across different stages, from laboratory animal to clinical, and licensed to the market [20]. This vaccine knowledgebase includes diverse vaccine information related to construction and vaccine-induced host responses, along with a compilation of cancer types. Additionally, CanVaxKB houses over 260 genes referred to as “canvaxgens”, which are associated with the development of cancer vaccine antigens.

The Vaccine Ontology (VO) is a community-based biomedical ontology that semantically represents various aspects of vaccine knowledge including vaccine type, vaccine formulation, and vaccine induced immune responses [21]. VO is built based on the upper-level Basic Formal Ontology (BFO) [22] and adhering to Open Biomedical Ontologies (OBO) Foundry principles. The OBO Foundry is a collaborative effort among ontology developers to build a set of interoperable and orthogonal ontologies in the biological and biomedical domains [23, 24]. In addition, several tools, such as OntoFox [25], OntoRat [26], ROBOT [27], Ontology Development Kit (ODK) [28], have been developed by the OBO Foundry community for automating ontology development. As a member of OBO library ontologies, the VO leverages existing terms from OBO Foundry ontologies, such as NCBI organismal classification (NCBITaxon) [29] for pathogenic organisms and vaccine targeted organisms, Protein Ontology (PRO) [30] for protein antigen, etc. Currently VO has over 8,200 terms that cover over 4,900 vaccines against over 200 pathogens and non-infectious diseases such as cancer. Our studies demonstrate that VO can be used to support data integration and advanced data analysis. Our previous VO research efforts have been focused on the usage of VO for representing vaccines against infectious diseases [31–33]. With the ongoing effort of CanVaxKB development, we have also used the VO to represent cancer vaccines [20].

In this paper, we systematically present our ontological modeling and analysis of cancer vaccines and associated knowledge using the VO and substantiated by detailed case studies. While the previous CanVaxKB paper [20] mentioned the usage of the VO for cancer vaccine standardization, that paper focused on the report of the CanVaxKB as a web knowledge and did on provide any details on the ontological modeling and applications. This paper provides such details and demonstrates that with certain extensions, the VO can serve as an effective ontology platform for cancer vaccine representation, which further offers unique features that facilitate the integration and analysis of cancer vaccine data. In addition, the constructed ontology development pipeline enabled automating cancer vaccine addition and edition in the VO.

## Methods

### Cancer vaccine data collection and annotation

Data for ontology representation was sourced primarily from CanVaxKB, incorporating peer-reviewed literature from PubMed, clinical trial information from ClinicalTrials.gov, and gene and taxonomy from NCBI [20]. This dataset includes diverse data types such as vaccine, target cancer, antigens (gene/protein ID), administration route, preparation methods, and developmental status. These details were standardized by aligning them with terms from established ontologies like the Vaccine Ontology (VO), Human Disease Ontology (DOID), and NCBI’s organismal classification, ensuring consistency across the dataset.

### Cancer vaccine ontology design pattern generation

Utilizing the VO as the foundation for ontological cancer vaccine representation, ontology design pattern (ODP) specific to cancer vaccines was developed by following standard ODP development method [26]. The pattern enhanced the existing general vaccine design pattern with cancer-specific features.

### Cancer vaccine-specific ontology development

The cancer-specific Ontology Design Pattern (ODP) informed the structuring of cancer vaccine data into logical axioms within the ontology. Data was formatted into a ROBOT template file (.csv) and converted to OWL format using ROBOT’s template function [27], subsequently integrating this data into VO.

For incorporating external ontology terms (e.g., disease terms), we employed Ontofox tool [28] for term retrieval and alignment within VO’s structure. The ROBOT’s remove function [27] also facilitated the removal of terms in the source ontology imported from external ontologies to avoid possible inconsistency or conflict as they may have diverged from the original definitions [34].

Ontology visualization, reasoning, and querying were conducted using Protégé ontology editor [35], with ELK 5.0 providing reasoning support to ensure ontology consistency. Furthermore, the ROBOT extract function (STAR method) [27] was instrumental in deriving a focused Cancer Vaccine Ontology (CVO) view from VO.

The entire ontology development process, from template conversion to ontology release, was automated using a Makefile, streamlining the workflow and ensuring reproducibility.

### Data access, license, and display

Cancer vaccine-related VO data and documentation is available at GitHub: https://github.com/vaccineontology/VO/tree/master/CVO. All source codes are available at VO GitHub: https://github.com/vaccineontology/VO. The data is available with license CC BY 4.0.

### DL and SPARQL queries

Both Description Logic (DL) query and SPARQL query are effective ways to query for the knowledge stored in ontologies. DL query and SPARQL query scripts were developed for VO-based data analysis to address different use cases. The Protégé DL query interface was used as the platform for DL queries with running the ELK5.0 reasoner before executing the queries over cancer vaccine. The Ontobee SPARQL web server (https://sparql.hegroup.org/sparql/) was used for SPARQL data query.

### Ontological modeling of cancer vaccine clinical trial studies

An ontological model is widely used to formally represent a set of entities and their relations in a computer-readable format. The ontological modeling was applied to identify the main entities in the cancer vaccine clinical evaluation. The data for cancer vaccine evaluation were extracted from clinical trial studies based on the ontological model and used for further analysis.

## Results

In the following, the single quotation marks ‘’ are used to indicate an ontology term. The italics are used to refer to an ontology relation terms (i.e. object properties).

### Cancer vaccine data

A total of 677 cancer vaccines targeting 42 cancer types, along with associated data, were collected in the CanVaxKB database. To ensure consistent representation of cancer vaccine data, we mapped the vaccines, vaccination routes, and vaccine platforms to VO, vaccine organisms and pathogens to NCBITaxon, and cancer types to the Human Disease Ontology (DOID) [36, 37] and the Mondo Disease Ontology (MONDO) [38] when the terms are not available in DOID.

Of the 42 cancer types, 39 were found in the DOID. For the three missing cancer types, two were identified in MONDO and we imported terms aligned with DOID in VO. One cancer type, ‘HPV associated cancer’ was not available in any OBO ontologies. ‘HPV associated cancer’ was present in VO as “A cancer that is caused by human papillomavirus (HPV) infection. HPV infection can lead to six types of cancer including anal, cervical, oropharyngeal, penile, vaginal, and vulvar cancer.” with logical axioms:

‘HPV associated cancer’ = (equivalent to: ) cancer and ‘*disease has basis in*’ some ‘HPV infection’.

Five vaccination routes and four vaccine platform types associated with cancer vaccines have been defined in VO. All vaccine organism and pathogen types were available in the NCBITaxon. However, only a few cancer vaccines existed in VO. Most of them need to be added to VO.

### Ontological design pattern for cancer vaccines

Traditionally, vaccines are developed to prevent or treat infectious diseases caused by pathogens such as bacteria, viruses, and protozoa [39]. Recently, there have been increasing studies in developing vaccines for diseases not typically caused by pathogens, such as cancer, allergy, and autoimmune diseases. To model cancer vaccines, we initially defined the term, ‘cancer vaccine’ in VO, as a vaccine that prevents or treats cancer. Logically, ‘cancer vaccine’ is equivalent to:

‘vaccine’ and ‘*immunizes against disease*’ some ‘cancer’.

This implies that any vaccine capable of immunizing against cancer is classified as a cancer vaccine.

In ontology, shortcut relations are often used to simplify and streamline complex relations among entities, making the ontology easier to understand and use. These shortcut relations are usually defined and only accessible in the ontology; however, their usages are often important for simplification, enhanced clarity, improved usability, and efficiency. In the VO, three key shortcut relations have been defined to link vaccines to critical entities associated with vaccines:

‘*immunizes against disease*’: a shortcut relation between vaccine and disease (e.g., cancer) that the vaccine can prevent or treat.

‘*immunizes host’*: a shortcut relation between vaccine and the organism that assumes the ‘vaccine host role’ is the target of a vaccine administration.

‘*immunizes against pathogen*’: a shortcut relation between a vaccine and a pathogen wherein the vaccine targets against the pathogen in the immunization process.

**Fig. 1:**
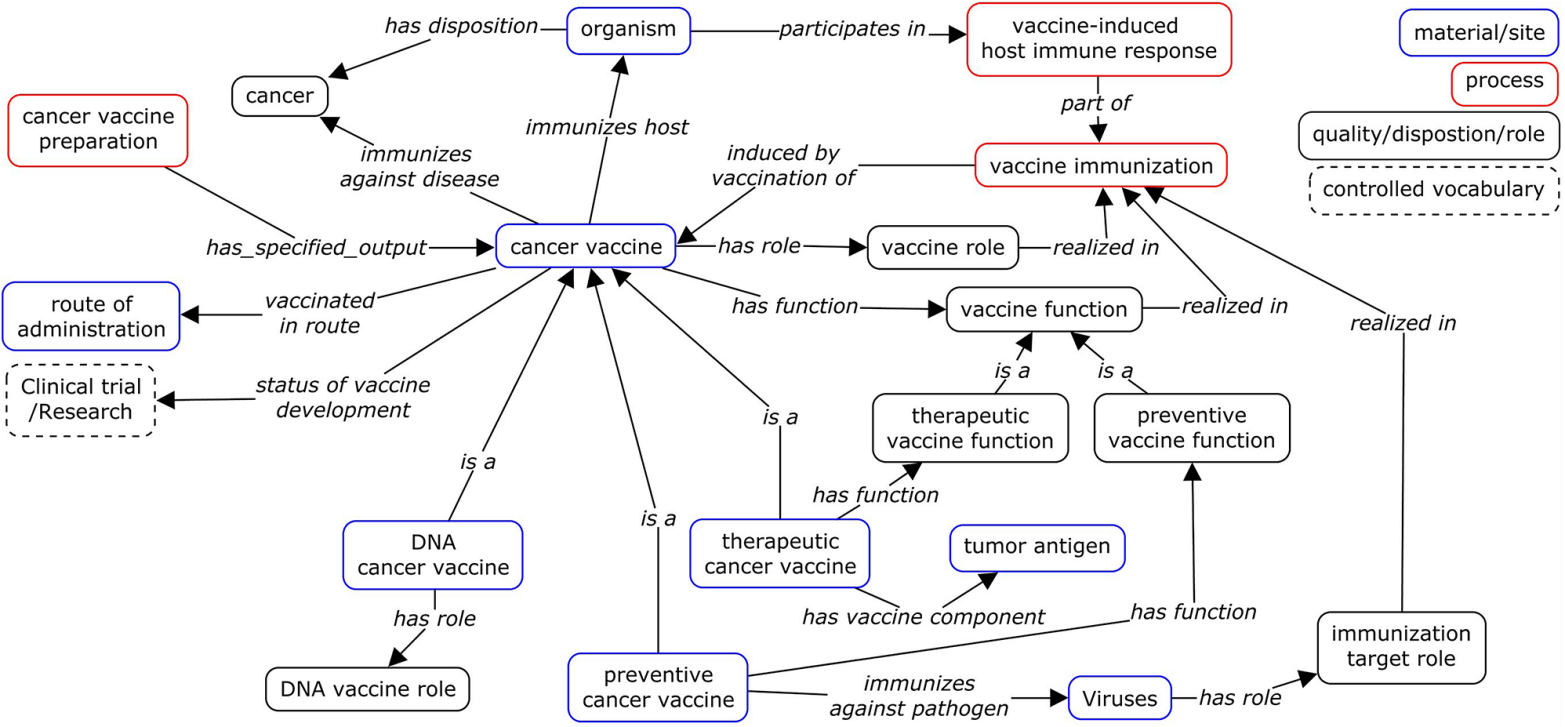
Cancer vaccine ontology design pattern (ODP). The pattern represented the collected cancer vaccine data (shown in box as VO classes) and the relations (as VO object properties) between them

We adopted the existing vaccine design pattern and extended it to create the cancer vaccine specific ontology design pattern (ODP) shown in Fig. 1. Two object properties ‘*immunizes against disease*’ and ‘*immunizes host*’ are used to represent the relations between a vaccine and a disease and a host, respectively. The relation *‘immunizes host’*, a shortcut linking a vaccine and the immunized organism assuming the ‘vaccine host role’. However, ‘vaccine host role’ in VO is a subClass of ‘host role’ from the Ontology for Biomedical Investigations (OBI) [40]. In OBI, ‘host role’ is defined as:

‘host role’ = def: A role played by an organism that provides nourishment, shelter, or a means of reproduction to another organism within the organism.

The above ‘host role’ only applies for organisms such as bacteria and protozoa. However, such a ‘host role’ may not be correct for cancer vaccines since most cancer vaccines do not deal with two organisms as seen in the ‘host role’ definition. To address this issue, we redefine the ‘vaccine host role’ in VO under the BFO: role (instead of under ‘host role’) specifically for the host of vaccine. Note that unlike the ‘host role’ definition provided above, the host target in our new term is vaccine, a foreign material entity that does not have to be another organism. The VO term ‘vaccine host role’ is now defined as follows:

‘vaccine host role’ = def: A role that inheres in an organism that hosts a vaccine being administered into the organism.

Different from traditional infectious disease vaccines, the relation ‘*immunizes against pathogen*’ does not apply to most cancer vaccines, as pathogens do not directly cause cancer. However, some virus infections can lead to cancer. For example, HPV infection can cause cervical cancer and other five types of cancer including anal cancer, oropharyngeal cancer, penile cancer, vaginal cancer, and vulvar cancer [10]. Consequently, anti-virus infection cancer vaccines have been developed to immunize against virus infection, thereby preventing associated cancer. For example, HPV cancer vaccine targets HPV to prevent the cancer caused by HPV infection [9–11].

Therapeutic cancer vaccines primarily target tumor antigens to elicit recognition and elimination of tumor cells by inducing the host immune response. For example, ‘Carcinoembryonic Antigen Peptide-1 Vaccine’ is a cancer vaccine that contains carcinoembryonic antigen, a tumor antigen, obtained from cancer cells which stimulates an immune response against tumor cells [41].

Figure 1 (bottom right) represents the ontological model of both preventive and therapeutic cancer vaccines. We defined specific ontology terms for these cancer vaccine in VO as follows:

‘preventive cancer vaccine’ = def: A cancer vaccine that prevents cancer development associated with viral infections.

‘therapeutic cancer vaccine’ = def: A cancer vaccine that aims to eliminate or control tumor cells by recognizing the tumor cells and stimulating the immune system via tumor antigens.

‘tumor antigen’ = def: An antigen expressed by the tumor cells, which may be exclusively present on tumor cells or overexpressed on them.

Further details on tumor antigen-related description and modeling are provided in the subsequent section.

Other features, such as vaccine platforms, vaccine administration routes, vaccine development status, associated with cancer vaccines are also represented in Fig. 1 (below). The models of vaccine administration routes and platforms are adopted from traditional pathogen vaccines [32]. A vaccine platform refers to the underlying technology or approach used to develop vaccines. Different vaccine platforms employ distinct strategies to stimulate the immune system and induce immunization against diseases. In VO, we modeled a platform based on the characteristics of primary material used in a vaccine. For instance, for vaccines containing pathogenic organisms, ‘organismal quality’, such as ‘organism inactivated’, ‘vaccine organism live attenuated’, describes the quality of organisms used. Otherwise, a specific ‘vaccine role’, such as ‘DNA vaccine role’ and ‘RNA vaccine role’, are used to differentiate vaccine prepared material (e.g. DNA, RNA). The ‘vaccine role’ is acquired in the vaccine preparation process and then realized in vaccine immunization. This model enables automated vaccine classification based on the platform.

Figure 1 illustrates the comprehensive process of ‘vaccine immunization’, wherein a ‘cancer vaccine’ is administered to an organism through a specified ‘route of administration’. This introduction stimulates the organism’s immune system, resulting in a ‘vaccine-induced host immune response’, and contributing to the prevention or treatment of cancer.

### Ontology modeling of cancer vaccine antigens and canvaxgens

Tumor antigens play a crucial role in stimulating therapeutic immune responses against cancers. Various types of cancer vaccine antigens exist, categorized based on the target and delivery methods (see Table 1). Shared tumor antigens are common across many patients [42], while personalized tumor antigens are unique to an individual’s tumor [43]. Anonymous tumor antigens involve either In Situ antigen-presenting cell (APC) colocalized vaccines [44, 45], which colocalize antigens with antigen-presenting cells (APCs) at the tumor site, or ex vivo cell vaccines, where this process occurs outside the body [10, 46]. Additionally, dendritic cell vaccines utilize dendritic cells to present tumor antigens to the immune system [5, 47]. Each category of cancer vaccine employs distinct strategies to engage the immune system, providing a comprehensive overview of the various approaches in cancer vaccine development and cancer immunotherapy.

**Table 1:** Overview of various approaches in cancer immunotherapy

Antigen type	Vaccine function	Vaccine subtype	Examples
Viral antigens	Preventive	Targeting Viral Infections	HPV Vaccine, Hepatitis B Vaccine
Shared tumor antigens	Therapeutic	Shared Antigen Vaccines	Provenge (Sipuleucel-T) for prostate cancer
Personalized tumor antigens	Therapeutic	Personalized Antigen Vaccines	NEO-PV-01 (personalized neoantigen vaccine)
Anonymous tumor antigens	Therapeutic	In situ APC Colocalized Vaccines	IVAC MUTANOME (personalized vaccine based on tumor mutanome)
Anonymous tumor antigens	Therapeutic	Dendritic Cell Vaccines	DCVax-L for glioblastoma
Anonymous antigens	Therapeutic	Ex vivo Cell Vaccines	GVAX

Efforts in cancer vaccine development focus on identifying and utilizing tumorigenic peptides as shared or personalized antigens (see Table 1) for effective vaccine development. Typically, cancer vaccines do not utilize entire genes or proteins as vaccine antigens due to the potential risks of severe adverse events, such as autoimmunity and severe diseases. Instead, cancer vaccine development often employs fragments of genes and proteins. To address the challenge of analyzing these fragments effectively without broader context, we introduce the concept of ‘canvaxgen’:

canvaxgen = def: A gene expressing a protein, either partially or entirely, serving as the antigen within a specific cancer vaccine.

**Fig. 2:**
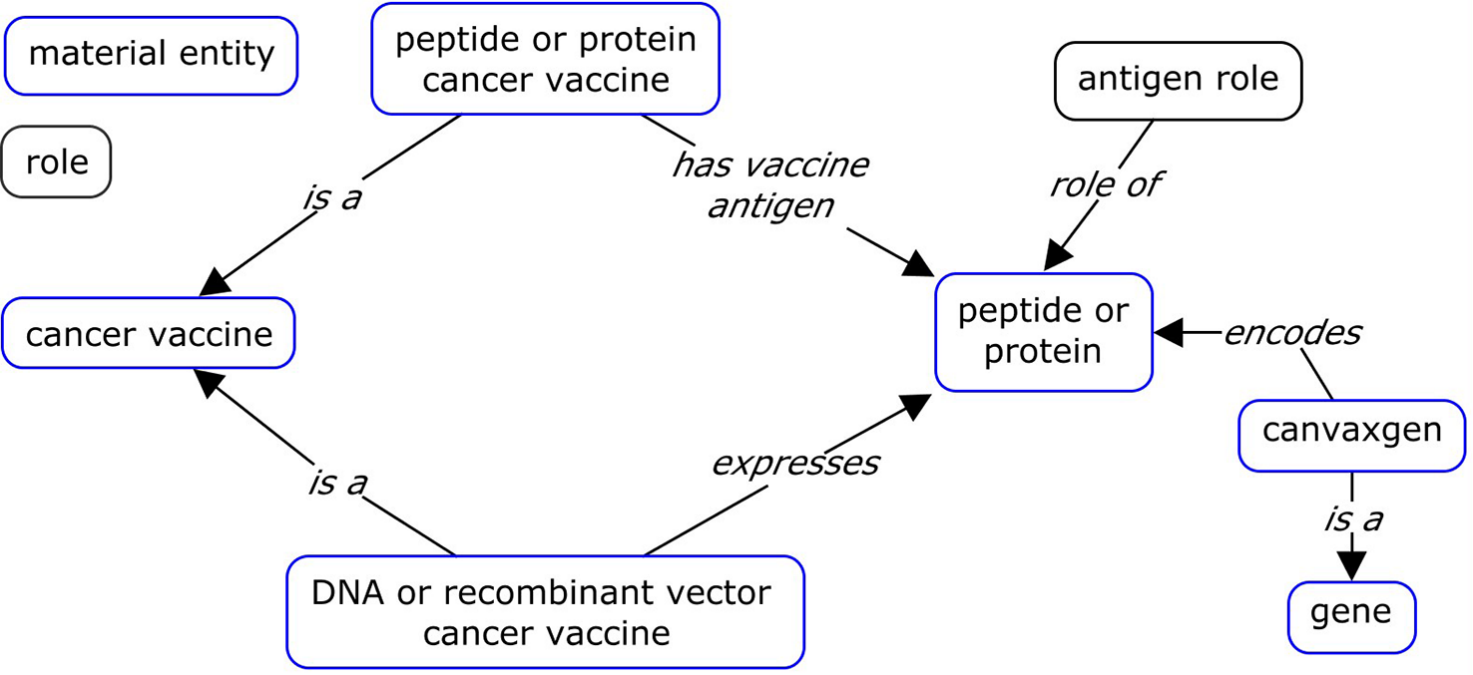
Ontological modeling of canvaxgens. The model illustrated the distinction between canvaxgen and antigen

It is important to distinguish canvaxgen from cancer vaccine antigen. Cancer vaccine antigens are specific proteins or peptides in cancer cells, which are targeted by the immune system following vaccination. Whereas canvaxgen covers genes responsible for expressing antigenic proteins or peptides within proteins, aiding in cancer vaccine development and analysis in cancer vaccine development and analysis (see Fig. 2).

As described in our recent CanVaxKB paper [20], we have identified 263 canvaxgens. Analyzing these genes using the Gene Ontology (GO) enrichment method revealed enriched features such as host-virus interactions of virus genes associated with cancer, cancer pathways, and tumor antigens [20]. This approach provides valuable insights that may not be accessible through a sole focus on peptide level.

### Ontology development based on cancer vaccine ontology design pattern (ODP)

We added 704 cancer vaccine terms to the VO using the methods described in the Methods section. To ensure reproducible and consistent representation of cancer vaccine terms, we applied the cancer vaccine ODP to construct the cancer vaccine term ROBOT template in CSV format file (https://github.com/vaccineontology/VO/blob/master/src/templates/cancer_vaccine.csv*).* The ROBOT tool [27] was then employed to generate cancer vaccine terms in an OWL file. Reused OBO Foundry ontology terms such as disease, host/pathogen organisms were extracted from the source ontologies such as DOID, MONDO, and NCBITaxon using OntoFox tool [25]. These OWL files were merged in VO.

**Fig. 3:**
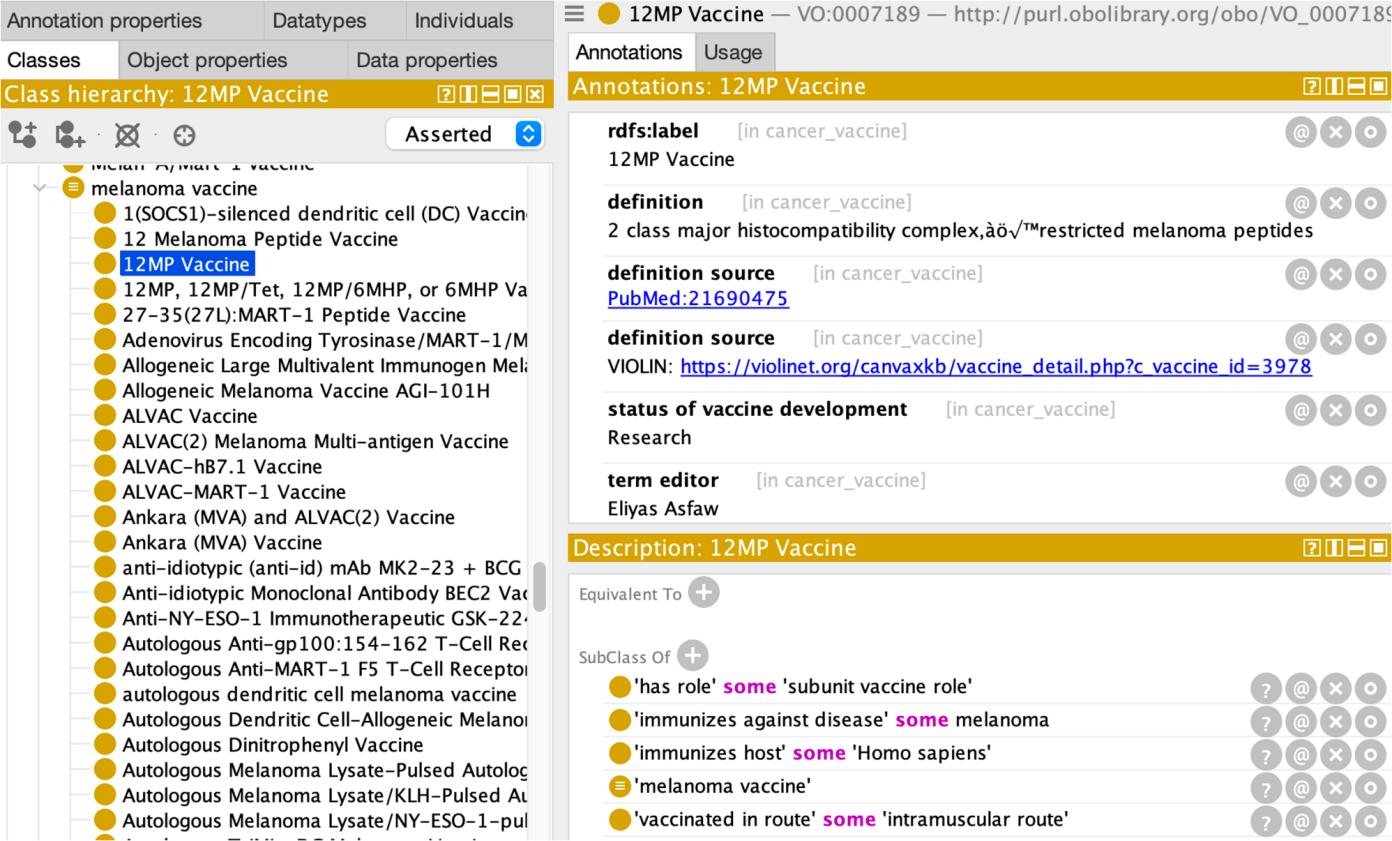
Protégé screenshot of a specific cancer vaccine in VO. The figure shows a specific cancer vaccine ‘12MP vaccine’ in VO that was generated based on ODP. It includes the term label, definition, definition source, status of vaccine development status in addition to logic axioms representing targeted organism and cancer type, vaccine platform, and vaccine administration route

Entities associated with cancer vaccines shown in the ODP were implemented as logic axioms of a cancer vaccine in VO. Figure 3 provides an example of a specific vaccine ‘12MP vaccine’ implemented based on the ODP. Specifically, this vaccine:

‘*is a’* ‘melanoma vaccine’

*‘immunizes against disease’* some melanoma

*‘immunizes organism’* some ‘Homo sapiens’

*‘has role’* some ‘subunit vaccine role’

*‘vaccinated in route’* some ‘intramuscular route’

In addition to the representing relations shown in the ODP, we added annotation properties, such as ‘definition source’ to include vaccine reference(s) and the CanVaxKB website link associated with a specific vaccine.

Cancer vaccines constitute a small portion of vaccines in VO. To facilitate working with a cancer vaccine only ontology, we generated the cancer vaccine view (cvo.owl) by retrieving all cancer vaccines and associated axioms from the full version VO. The cancer vaccine view is available on: http://purl.obolibrary.org/obo/vo/cvo.owl. As of March 12, 2024, the cvo.owl contains 928 classes with 681 specific cancer vaccines and 23 cancer vaccine terms such as ‘lung cancer vaccine’, ‘melanoma vaccine’ for term organization. It includes 744 VO specific classes and reused 154 classes from external ontologies (15 BFO classes, 92 DOID classes, 3 GO classes, 9 IAO classes, 2 MONDO classes, 26 NCBITaxon Classes, 15 OBI classes and 3 PATO classes). This CVO view also has 16,003 axioms including 3,706 logical axioms.

Additionally, a Makefile was generated to provide instructions to automate the above ontology development workflows, including ‘modules’ to convert template to OWL file, ‘imports’ to retrieve terms from external source ontologies and clean the axioms, ‘all’ to build a merged and inferred version of VO and generate OBO Foundry principle checking report, and ‘views’ to generate cancer vaccine view.

### Analysis of cancer vaccines based on cancer disease types using DL query

**Fig. 4:**
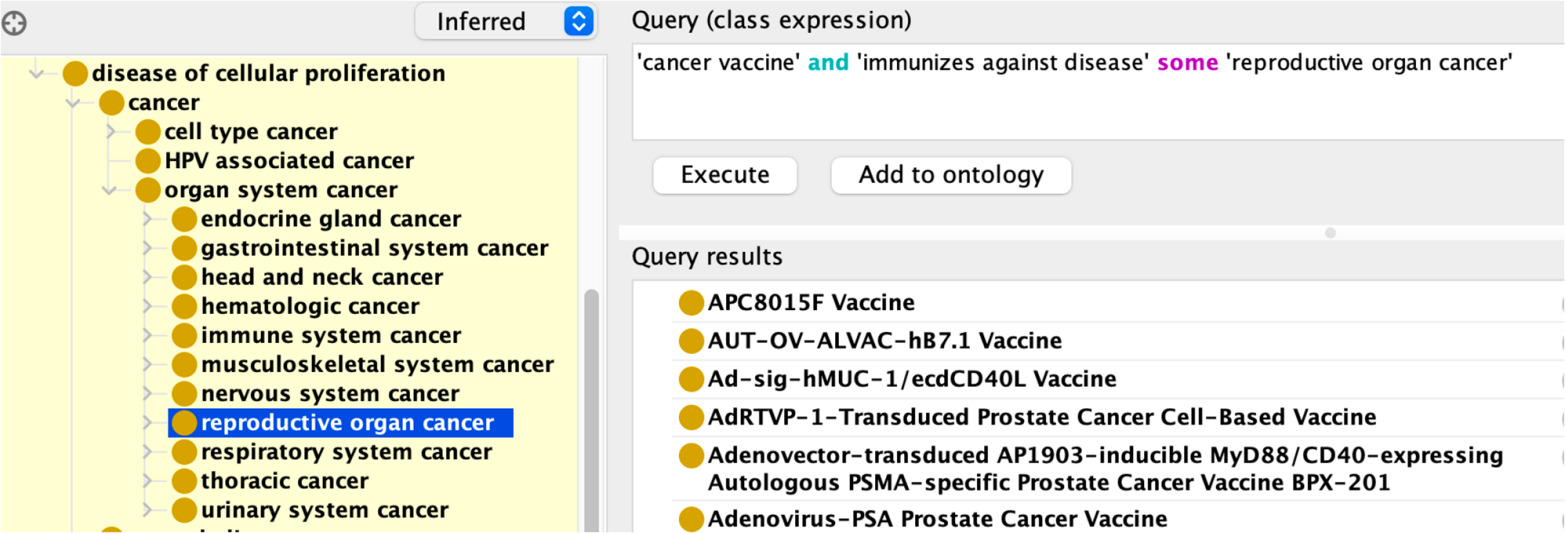
Protégé screenshot of a DL query in VO. The DL query is used to retrieve all the cancer vaccines that immunize against reproductive system cancer. The query is shown at the top right of the screenshot, while the results are shown at the bottom right

350 cancer vaccines target cancer occurring in specific anatomical entities, with some vaccines targeting multiple types of cancer occurring in different anatomical sites. For example, ‘RAS Peptide Cancer Vaccine’ targets RAS peptide-specific anti-tumoral T-cell cytotoxic immune response, inhibiting cancer in colon, pancreas, and lung [48]. Figure 4 illustrates a DL query example that was used to retrieve all cancer vaccines targeting ‘reproductive organ cancer’ based on ‘immunizes against disease’ logic axioms.

**Fig. 5:**
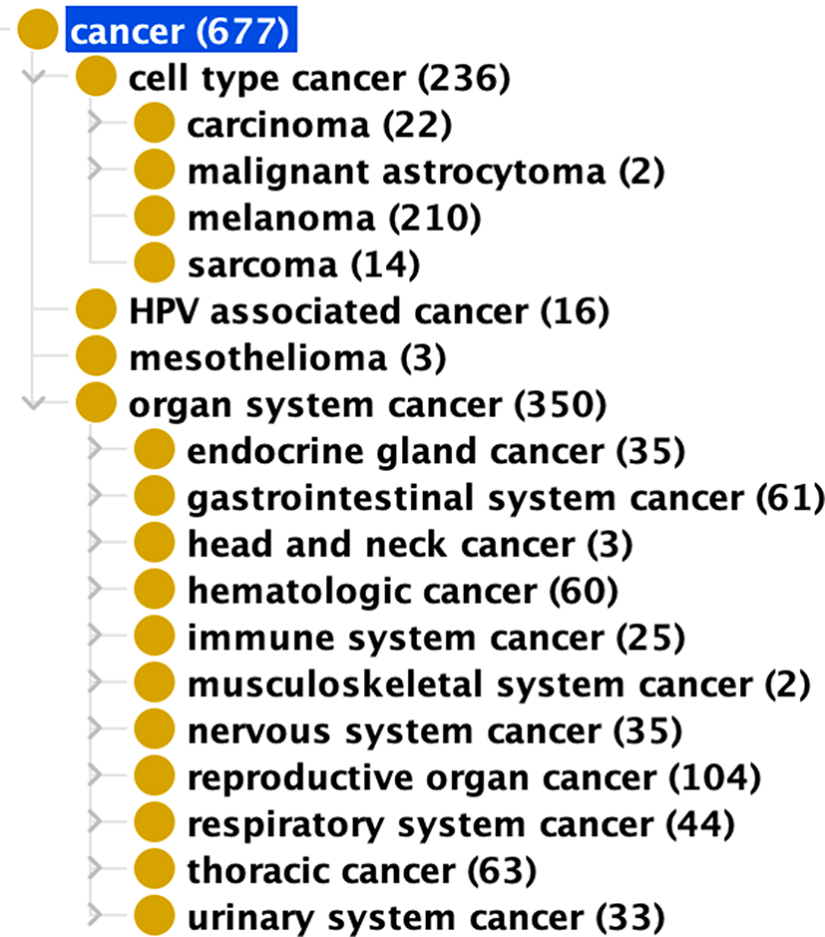
Hierarchical classification of cancers and cancer vaccines for specific cancers. The cancer hierarchy was generated using OntoFox tool and visualized using Protégé, and the numbers next to the cancer types were obtained using DL query for VO

These 350 cancer vaccines target 11 types of ‘organ system cancers’ defined in Disease Ontology (DOID), including 104 vaccines against reproductive organ cancers, 61 vaccines against gastrointestinal system cancers, and 60 vaccines against hematologic cancers, etc. (Fig. 5).

According to DOID, cancer is classified not only by anatomical entities (i.e. ‘organ system cancer’) but also by the type of cell from which it is derived (i.e. ‘cell type cancer’). DOID contains 4 categories under the ‘cell type cancer’. Based on cell types, CVO has 236 cancer vaccines, including 210 melanoma vaccines, 22 carcinoma vaccines, 14 sarcoma vaccines, and 2 malignant astrocytoma vaccines (Fig. 5). Melanoma is a skin cancer that starts in the melanocytes [49]. Carcinoma is a malignant neoplasm of epithelial cell origin of the internal or external lining of the body [50]. Astrocytoma is a cancer of astrocytes that support and connect nerve cells in the brain and spinal cord [51]. A sarcoma is a malignant tumor that arises from cells of mesenchymal origin (e.g., bone, muscle, fibrous tissue) [52].

Figure 5 summarizes all cancer vaccines classified based on the cancer disease hierarchy. The number of the vaccines obtained using DL query based on *‘immunizes against disease’* targeted on different cancer types.

### Analysis of cancer vaccines based on vaccine platform using SPARQL query

Cancer vaccines can be prepared by different vaccine platforms including subunit vaccine, DNA vaccine, recombinant vector vaccine, and live attenuated vaccine. These platforms are general vaccine development methods not unique for cancer vaccines. The vaccine platforms were defined using equivalent axioms that allowed the vaccines to be classified based on vaccine platforms in addition to other vaccine features such as immunized disease, targeted pathogen, etc. For example, ‘DNA vaccine’ is defined as:

def: A vaccine that is composed of a plasmid vaccine vector (a circular double stranded DNA molecule) containing the whole of parts of genes encoding one or more vaccine antigen proteins.

equivalent to:

‘vaccine by platform type’ and *‘has role’* some ‘DNA vaccine role’

We developed SPARQL query scripts to retrieve cancer vaccines of interest including those cancer vaccines based on specific vaccine platforms. For example, the following query is used to obtain all DNA cancer vaccines. The SPARQL query can be used to retrieve cancer vaccines of interest. For example, the following query is used to obtain all DNA cancer vaccines:

PREFIX cancer_vaccine:<http://purl.obolibrary.org/obo/VO_0000177>

PREFIX has_role: <http://purl.obolibrary.org/obo/RO_0000087>

PREFIX DNA_vaccine_role: <http://purl.obolibrary.org/obo/VO_0000623>

SELECT distinct? vaccine_label? vaccine.

FROM < http://purl.obolibrary.org/obo/merged/VO>

WHERE.

{

?vaccine rdfs: label? vaccine_label .

?vaccine rdfs: subClassOf* cancer_vaccine: .

?vaccine rdfs: subClassOf? vaccine_restriction .

?vaccine_restriction owl: onProperty has_role:;

owl: someValuesFrom DNA_vaccine_role: .

}

Running on the Ontobee SPARQL endpoint, the above script returned 86 DNA cancer vaccines with vaccine name and IRI, such as ‘Cancer DNA vaccine p.DOM-AH1 encoding fragment C’ (http://purl.obolibrary.org/obo/VO_0004425*)*, ‘Melanoma DNA vaccine pN4a-MAGE-1-GM’ (http://purl.obolibrary.org/obo/VO_0004435*)*, and ‘Lung metastasis DNA vaccine pCEP4-MUC1 encoding MUC1’ (http://purl.obolibrary.org/obo/VO_0004437*).* These DNA vaccines contain DNAs that encode different proteins or protein fragments and target different cancer types. While relational database-supported web queries may provide a more user-friendly interface, using SPARQL queries allows us to efficiently query ontology-stored knowledge using the standard query language from an ontology triple store.

### Ontological modeling cancer vaccine clinical trial studies and applying to melanoma vaccine clinical trial data analysis

Cancer vaccines aim to stimulate a robust host response for effective immunotherapy and immunoprevention. The main goal of the host response is to achieve a sustained and high level of immune response through cancer vaccine, which includes immunity to specific antigenic epitopes [53]. About 70% of vaccines collected in CanVaxKB are in the development stage of clinical trials. However, it can be a challenge in evaluating the host responses to cancer vaccines, especially in clinical trials involving human subjects.

Response Evaluation Criteria in Solid Tumors (RECIST) is a standardized criteria by multiparty validation to assess changes in solid tumors. It is also commonly used as a means of evaluation in cancer immunotherapy and cancer vaccine outcomes [54, 55]. RECIST has adopted twelve criteria to quantify tumor growth and defines terms like complete response (CR), partial response (PR), stable disease (SD), or progressive disease (PD) to further classify tumor clinical trial response [56]. The RECIST criteria could also be used to study the associations with tumor long-term outcomes [54]. Therefore, we have adopted RECIST for cancer vaccine standardization outcome measurements.

**Fig. 6:**
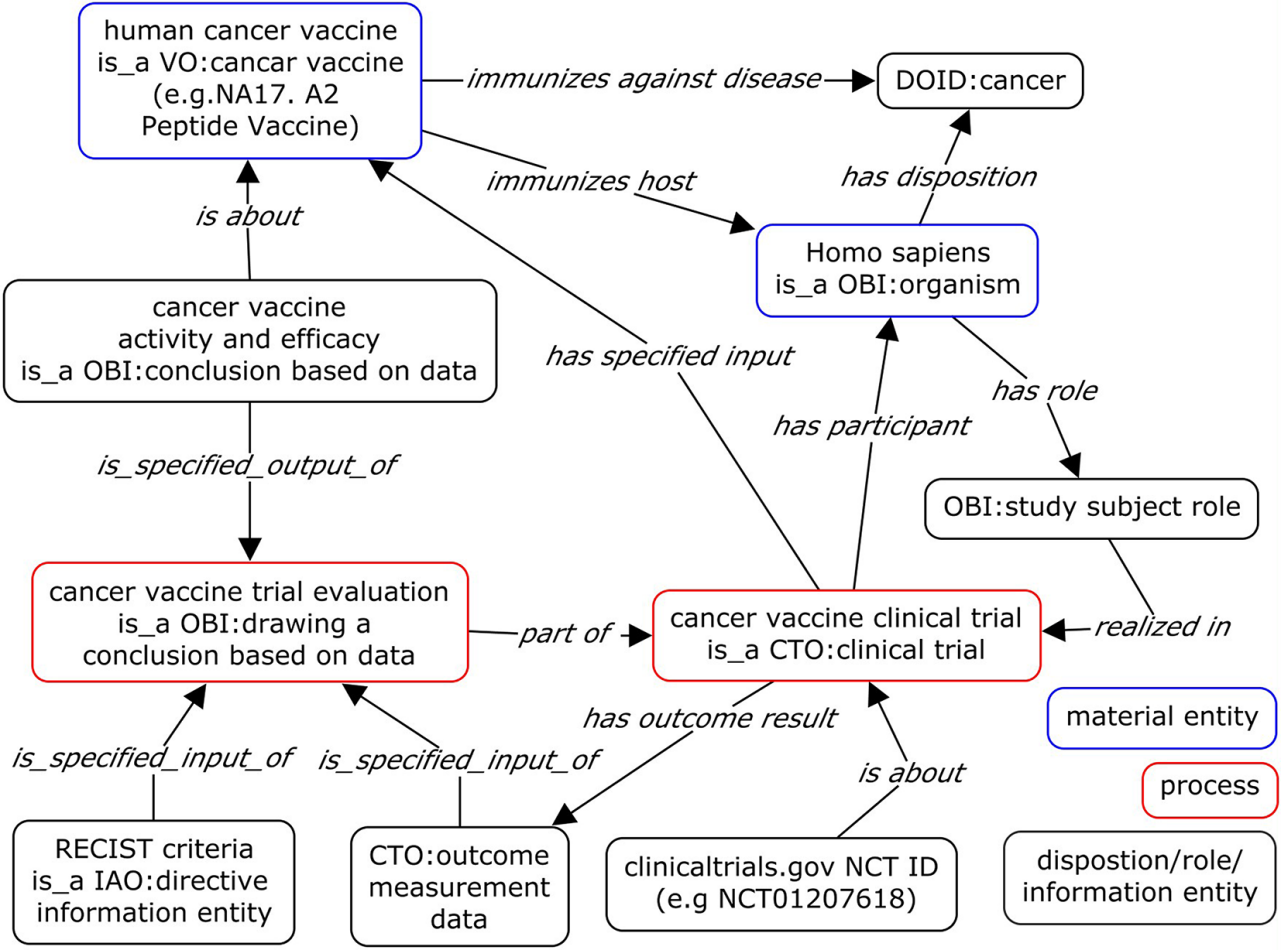
Ontological modeling of cancer vaccine clinical trials. The model represented main processes in a clinical trial study and showed how cancer vaccine activity and efficacy were evaluated using RECIST criteria. The terms with prefix indicate the source of the ontologies. All ontologies used in the model are OBO Foundry ontologies. CTO: Clinical Trial Ontology, DOID: human Disease Ontology, IAO: Information Artifact Ontology, OBI: Ontology for Biomedical Investigations, and VO: Vaccine Ontology

OBO Foundry ontologies were used to represent main components and their relations in cancer vaccine clinical trials including assessment of cancer vaccine efficacy according to RECIST criteria. A cancer vaccine clinical trial involves participants, who are human study subjects with cancer and receive cancer vaccines as part of the trial. Each clinical trial study has a clinical trial identifier commonly used to reference the specific clinical trial on clinicaltrials.gov. The clinical trial generates outcome measurement data that are assessed according to RECIST criteria to draw conclusions regarding the efficacy of the cancer vaccine against the cancer (Fig. 6).

**Fig. 7:**
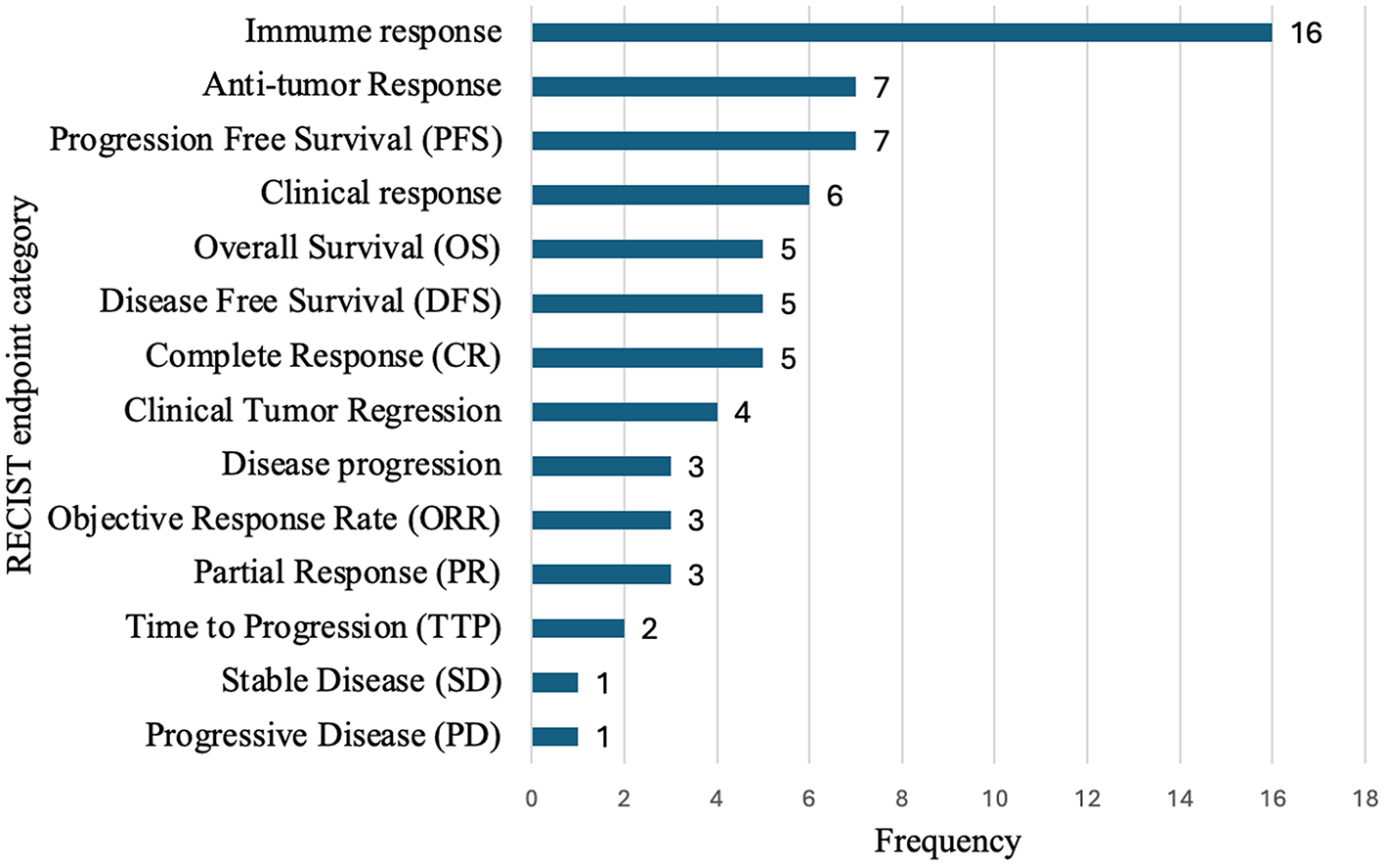
Occurrence of different patient outcome endpoints measurements of melanoma vaccines. The results generated from clinical trial endpoints analysis of 53 annotated melanoma cancer vaccine data. The endpoints were measured and categorized based on RECIST. The numbers located next to the bars indicated the frequency occurred for corresponding endpoint

The ontology model was applied to collect patient outcome measurements after melanoma vaccine treatment from the clinical.gov. Then the vaccine efficacies were analyzed using RECIST criteria. The RECIST endpoints were assigned through manually evaluated patient outcomes of 83 melanoma vaccine clinical trial studies. Figure 7 shows the frequency of the endpoint measurements. The results of the assessment illustrate the effect and activity of cancer vaccines. For example, a total of 6 cases of Anti-tumor response and 13 cases of immune response were reported among the 83 annotated melanoma clinical trial studies. This result reflects that the accessed melanoma vaccines work effectively on immune reactions to identify or attack abnormal cancerous cells and stimulate a cytotoxic T-cell reaction. Also, the immune system’s ability to generate a strong response significantly influences the likelihood of achieving a favorable outcome defined by RECIST.

The use case demonstrated that ontology models can guide the cancer vaccine clinical trial data collection for vaccine efficacy analysis. Standardization of the data and representation details of RECIST criteria using ontology may potentially enable the automated analysis of vaccine immune response in the future.

## Discussion

This study contributes to multiple facets of cancer vaccine. First, we have developed a comprehensive ontological model for cancer vaccines, encompassing various aspects and established a self-contained ontology design pattern (ODP). Secondly, we have implemented a cancer vaccine ontology development pipeline, exemplified by the creation of all the cancer vaccines in CanVaxKB in an ontology format (i.e., cancer vaccine ontology or CVO), which is also part of the community-based Vaccine Ontology (VO). Thirdly, we have applied the CVO to systematically analyze different dimensions of the cancer vaccines.

To our knowledge, this paper represents the first comprehensive ontological modeling for cancer vaccines. The significance of such modeling is substantial given the increasing accumulation of data on cancer vaccines. Ontological models play a crucial role in standardizing and integrating cancer vaccine data from diverse sources. In addition to traditional vaccines, we have specifically modeled aspects unique to cancer vaccines, focusing on vaccine functions and vaccination mechanisms.

The ontological modeling of cancer vaccines has facilitated the construction of cancer vaccine data in a tubular template for automatic generation of ontology terms. The established cancer vaccine ontology development pipeline enables the efficient and consistent addition of new cancer vaccines. Moreover, the template-driven ontology term generation approach allows for easy editing of existing terms when the ODP is modified or extended by updating the tubular template and rerunning the OWL conversion script.

Modeling vaccine platforms remains a significant challenge due to the multitude of factors involved, such as cancer type, characteristics of the targeted tumor antigens, intended delivery method, desired immune response. Currently we have only incorporated well-established vaccine platforms based on vaccine prepared material. However, newer platforms developed for cancer vaccines, such as dendritic cell-based vaccines, tumor cell-based vaccines, etc., are not yet represented in VO. Enhancements to the current model are necessary to accommodate various platforms and consider more critical aspects related to them. Additionally, while tumor antigens are essential components of cancer vaccines, satisfactory ontological representation of those antigens remain unresolved, and further work is planned to address this in the future.

Regarding disease annotations, we primarily utilized DOID, a human specific disease ontology [36, 37] to annotate cancer types. Although cancer vaccines are mainly aiming to protect or prevent human cancer, many vaccines are developed and tested using animal models. While considering using MONDO for disease annotations due to its inclusion of multi-species disease terms, we encountered challenges related to distinguishing between disease and disorder concepts. OBO Foundry ontologies define these concepts separately, and maintaining this distinction is essential for clarity in ontology. Disorders are the physical basis of a disease. The ‘disease’ is a disposition to undergo pathological processes that exist in an organism because of one or more disorders in that organism [57]. The ‘disorder’ is a material entity which is clinically abnormal and part of an extended organism [57]. Thus, we predominantly relied on DOID in this study, but we aim to collaborate with OBO Foundry developers to resolve these issues and incorporate cross-species disease terms eventually.

While our current analysis has focused on gene level, we observed that many peptides within specific protein encoded by canvaxgens are frequently utilized in therapeutic cancer vaccine development. This phenomenon reflects the personalized nature of immunotherapy, where different patients may have mutations in the same canvaxgen. Notably, multiple TP53 mutations have been identified in cancer vaccines using the TP53 as the canvaxgen [58].

In the future, we intend to search deeper into various aspects of cancer vaccines using ontology-based approaches. This includes finalizing the implementation of canvaxgen and cancer vaccine antigens and associating them with corresponding cancer vaccines in VO. It is possible to integrate the modeling of genetic mechanisms of action of cancer vaccine antigen genes (i.e., canvaxgen) using ontologies such as the Gene Ontology. It is also possible to refine our VO ontological representations from different aspects. For example, the similarities and differences between ‘vaccine function’ and ‘vaccine role’ can be further discussed and refined. Current definition of ‘vaccine role’ appears very broad and encompass all types of interventions. However, the ‘vaccine function’ can be more specific and may need to be prioritized. We will continue to collect and annotate more cancer vaccines, leverage ontology hierarchy and logic axioms to enhance data exploration and analysis within CanVaxKB. Additionally, we will employ bioinformatic analyses to refine existing patterns and identify new insights among cancer vaccines.

## Data Availability

The cancer vaccine ontology view is available at: github.com/vaccineontology/VO/tree/master/CVO. Cancer vaccine-related ontology data is available at Vaccine Ontology (VO) GitHub: github.com/vaccineontology/VO.

## Abbreviations

VO: Vaccine Ontology
CVO: Cancer Vaccine Ontology
OBO: Open Biomedical Ontologies
NCBITaxon: NCBI Organismal classification
PRO: Protein Ontology
DOID: Human Disease Ontology
MONDO: Mondo Disease Ontology
DL: Description Logic
HPV: Human Papillomavirus
RECIST: Response Evaluation Criteria in Solid Tumors
